# Towards a Contactless Stress Classification Using Thermal Imaging

**DOI:** 10.3390/s22030976

**Published:** 2022-01-27

**Authors:** Federica Gioia, Alberto Greco, Alejandro Luis Callara, Enzo Pasquale Scilingo

**Affiliations:** 1 Dipartimento di Ingegneria dell’Informazione, University of Pisa, 56122 Pisa, Italy; alberto.greco@unipi.it (A.G.); alejandro.callara@ing.unipi.it (A.L.C.); e.scilingo@ing.unipi.it (E.P.S.); 2 Research Center “E. Piaggio”, University of Pisa, 56122 Pisa, Italy

**Keywords:** thermal imaging, stress detection, wearable systems, support vector machine, contactless

## Abstract

Thermal cameras capture the infrared radiation emitted from a body in a contactless manner and can provide an indirect estimation of the autonomic nervous system (ANS) dynamics through the regulation of the skin temperature. This study investigates the contribution given by thermal imaging for an effective automatic stress detection with the perspective of a contactless stress recognition system. To this aim, we recorded both ANS correlates (cardiac, electrodermal, and respiratory activity) and thermal images from 25 volunteers under acute stress induced by the Stroop test. We conducted a statistical analysis on the features extracted from each signal, and we implemented subject-independent classifications based on the support vector machine model with an embedded recursive feature elimination algorithm. Particularly, we trained three classifiers using different feature sets: the full set of features, only those derived from the peripheral autonomic correlates, and only those derived from the thermal images. Classification accuracy and feature selection results confirmed the relevant contribution provided by the thermal features in the acute stress detection task. Indeed, a combination of ANS correlates and thermal features achieved 97.37% of accuracy. Moreover, using only thermal features we could still successfully detect stress with an accuracy of 86.84% in a contact-free manner.

## 1. Introduction

Thermal imaging is currently taking hold in psychophysiology for its great advantage of measuring skin temperature noninvasively, ecologically, and contact free [1]. Many changes in skin temperature are regulated by the interplay between the parasympathetic (PNS) and sympathetic (SNS) branches of the autonomic nervous system, i.e., blood redistribution, perspiration, muscle activation, and metabolism. For instance, the activation of the SNS can alter the vasomotility of subcutaneous vessels (e.g., vasoconstriction) with consequent changes in the skin blood perfusion [2,3], or may control the sweat gland activation modifying skin perspiration [4]. Accordingly, monitoring skin temperature may provide information on SNS activity and its dysregulation induced by factors such as chronic or acute stress.

After a stressful stimulus, the sympathetic activation results in a series of rapid sympathetically driven adaptation changes of ANS activity, whose correlates are reflected by several peripheral signals. Among these, the breathing rate increases promoting oxygenation, the blood flow is redirected to large muscles through blood vessels dilation, and the heart rate and blood pressure increase, while digestive functions, urination, and feeling of hunger are inhibited [5]. Although stress is a physiological phenomenon that is helpful in many demanding situations, acute stress episodes could affect the human physical condition and health leading to several symptoms ranging from headache to heart attacks or arrhythmias [6,7,8].

Typically, stress is monitored by means of questionnaires, psychometric tests, and interviews. However, these methods provide subjective measures and can lead to a wrong assessment of the stress level when the subject is not completely honest or able to answer. Accordingly, many studies have attempted to infer the psychological state of a subject in a more reliable and unbiased manner by analyzing ANS peripheral correlates [9]. Among these, the electrodermal activity (EDA) could provide a reliable metric of stress, and it has been used as a ground-truth to compare the performance of other signals [10,11,12,13]. However, in view of a more comprehensive ANS assessment, a combination of autonomic measures, such as respiratory (RESP), cardiac, and EDA signals, is often adopted in the scientific literature, by using multiple wearable devices [14,15]. However, such an approach may affect the subject’s natural behaviour both in laboratory settings and in real life scenarios mainly because of the presence of electrodes and cables. In this study, we contributed to the shift towards contactless stress monitoring by extending standard approaches to stress recognition with the aid of infrared thermography.

Thermal imaging unobtrusively monitors skin heat distribution mapping infrared (IR) radiation onto an image called a thermogram. So far, several physiological parameters have been extracted from the thermograms, such as breathing rate [16], cardiac pulse [17], and cutaneous blood perfusion [18]. In addition, information directly derived from the thermal time series has been analyzed in relation to mental states and emotional stimuli [19,20,21,22,23]. For example, the thermal variation of the nose tip has been used to discriminate stimuli with positive and negative valence [24], as well as in response to startling stimulus [25], but also to fear or stress [26,27,28,29]. Furthermore, thermal features from several facial regions have been used for statistical comparisons among different psychological states [1,30], and to classify emotional states [31,32]. In the stress-assessment context, stress elicited by the Stroop test was observed to induce an enhancement of blood flow in the forehead, supraorbital and frontal vessels, as indicated by different slopes of the blood volume signal obtained from the thermograms in baseline and stress condition [29].

We performed a systematic study to quantify the contribution of thermal imaging to a multisensor wearable system for stress recognition. In particular, we evaluated whether thermal features can improve the results obtained by a wearable system based on well-established physiological features extracted from EDA, HRV, and RESP signals in automatically and reliably recognizing stress. Moreover, we investigated the potential use of the contactless monitoring system in substitution of the wearable one. We followed a rigorous and robust methodology with the purpose of increasing the accuracy achieved by previous studies. At the same time, we included innovative facial regions (i.e., nasal septum, left and right forehead, and left and right chin) and thermal features (i.e., derivative of the mean temperature and its standard deviation). We compared the classification performance of a support vector machine with recursive feature elimination (SVM-RFE) classifier using different sets of physiological and thermal features. More specifically, we acquired physiological signals, i.e., electrocardiogram (ECG), EDA, and RESP along with thermal images from 25 healthy subjects performing a computerized/paced Stroop test. On this dataset, we conducted a preliminary statistical analysis comparing features extracted from the EDA, HRV, RESP, and thermal signals during resting and stressful conditions, with the aim of evaluating the effectiveness of our protocol in eliciting acute mental stress. Then, we evaluated whether thermal features improve stress/non-Stress classification accuracy when integrated with more standard physiological ones. Finally, we attempted to recognize stressful conditions by using thermal features alone. All of the details are reported in the next sections, organized as follows: in Section 2, we describe the protocol and acquisition devices, processing methods for each signal, feature extraction, statistical analysis, and classification algorithm. In Section 3 we show the experimental results and in Section 4 discuss our findings.

## 2. Materials and Methods

### 2.1. Study Population

A total of 25 healthy subjects (9 females, age = 27 ± 3.1 years) participated in this study. The experiments were approved by the “Bioethics Committee of the University of Pisa” (n. 15/2019), and each volunteer signed the informed consent. Subjects were asked not to drink coffee the day of the experiment to limit potential confounds due to vasomotor effects (e.g., increased/decreased vasomotility) [33]. For the same reason, they were also asked to avoid the consumption of alcohol and tobacco starting on the day before the experiment.

### 2.2. Experimental Protocol

**Figure 1:**
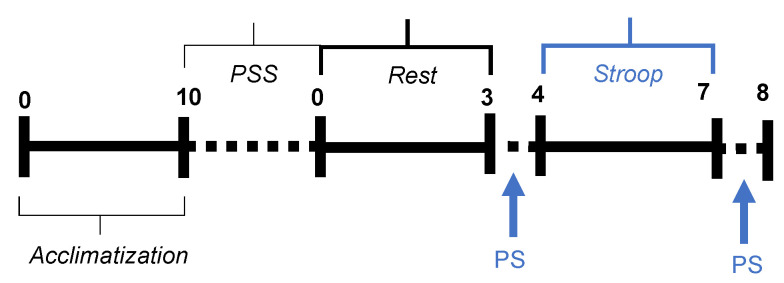
Protocol timeline. The dashed line indicates that the subject may take as much time as he/she needs to complete the task. PS indicates the moment when the subject is asked to report its perceived stress level.

The experimental timeline is reported in Figure 1. It consisted of three 3 min long sessions preceded by a 10 min long acclimatization period.

The first session represented a baseline condition during which subjects were resting with their eyes open. The second session aimed to induce mental stress by means of the Stroop test [34]. This latter is a well-known stressor that requires the fast resolution of two incongruous stimuli causing cognitive interference. More specifically, incongruent color/semantic-meaning words are presented to a subject who has to answer based on the color of the word. Here, we developed a computerized paced version of the Stroop test, in which the name of a color was presented in the middle of the tablet screen every two seconds, and the subjects had to press the button corresponding to the tint of the word before a new stimulus appeared. We included a counter, keeping track of the consecutive success as a motivational stressor. During the task, a ticking sound was being played in the background to mark the passage of time. Additionally, for each wrong/missing answer the counter was reset while an acoustic buzzer alerted the subject. Finally, before and after the *Stroop* session, subjects were asked to report their level of perceived stress on a Likert scale from 0 (not at all) to 10 (very stressed). The self-assessed perceived stress level served as a reference for our analyses.

All the instructions were presented to the subjects on a tablet. Accordingly, interactions with the experimenter were limited, preventing speech artefacts in the data and standardizing the conditions for all subjects. Additionally, each subject filled out the Perceived Stress Scale (PSS) questionnaire. The PSS is a standard psychological test for measuring the perception of stress, as the degree to which situations in one’s life are appraised as stressful [35].

The room temperature and humidity were monitored throughout the experiment by the EXTECH RH550 Humidity-Temperature Chart Recorder (Extech Instruments, Nashua, New Hampshire, U.S.) at a rate of 0.5 Hz. In particular, we allowed a maximal temperature variation within one experimental session of ±1 ${}^{\circ }$C and a humidity variation of ±5%. Indeed, a stable environment was required to ensure reliable relative measures of facial skin temperature. Moreover, since these variables could trigger body thermoregulatory responses not related to emotional stress, temperature and humidity were always in a comfortable range of 22 ${}^{\circ }$C to 28 ${}^{\circ }$C and 40% to 56%, respectively [36]. Finally, the subject was placed in a seated position at a fixed distance of 1 m from the camera, at maximum distance from the window in order to avoid direct sunlight, and with no direct ventilation. Taking this into account, we could ensure reliable relative measures without adding the influence of ambient temperature and humidity on facial area temperature. In addition, subjects were asked to remove corrective eyewear during the experiment, as glass is opaque to infrared light.

### 2.3. Data Acquisition

#### 2.3.1. Thermal Imaging

We acquired both thermal and RGB images of subjects’ faces. The skin temperature distribution was unobtrusively acquired through a FLIR T640 thermal camera (Teledyne FLIR LLC, Wilsonville, Oregon, U.S.), at a rate of 5 Hz. The camera had a resolution of 640 × 480 pixels, 24.6 mm lens, NETD < 0.04 mK @ +30 ${}^{\circ }$C and spectral range of 7.8–14 μm (long-wave infrared—LWIR). Additionally, standard RGB images were acquired at a rate of 30 Hz using a Logitech HD Webcam C270 (Logitech International S.A., Lausanne, Switzerland), 1280 × 720 pixels, vertically aligned and fixed to the thermal camera.

#### 2.3.2. Physiological Signals

We recorded EDA, ECG, and RESP using a BIOPAC MP 150 system (Biopac Systems, Inc., Goleta, CA, USA), at a sampling rate of 500 Hz. EDA was recorded placing two electrodes on the distal phalanx of the index and ring fingers of the non-dominant hand. ECG was recorded with 3 electrodes positioned below the right and left clavicle, and on the lower left chest. Finally, RESP was monitored with a piezoelectric chest belt.

### 2.4. Data Processing

#### 2.4.1. Thermal Processing

**Figure 2:**
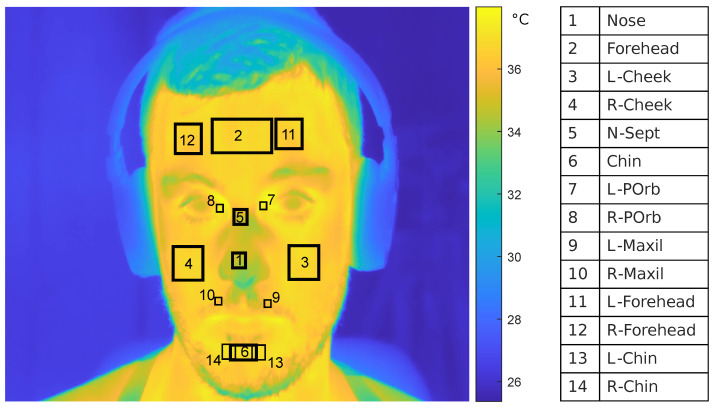
Facial ROIs’ names and corresponding locations.

We used thermal imaging to monitor the variation in facial temperature distribution during the experiment and RGB frames to identify anatomical landmarks of interest for the analysis of IR images. Accordingly, RGB and IR acquisitions were synchronized after downsampling the RGB video to 5 Hz (i.e., IR imaging sampling rate). Then, RGB and IR frames were spatially registered with an affine-2D transformation matrix estimated from a set of manually selected fiducial points in the first frame of the RGB and IR datasets, such as periauricular points, the corner of the eyes and the mouth. After RGB-IR spatiotemporal registration, we defined 14 regions of interest (ROIs): i.e., nose tip, forehead (central, right and left), cheek (right and left), nasal septum, chin (central, right, left), periorbital (right and left), and maxillary (right and left). A summary of these ROIs along with their corresponding location is represented in Figure 2.

For each subject, ROIs were automatically found with a two-step procedure. First, the ROI centres were identified through the intersection of lines connecting specific facial landmarks detected in the RGB frames with the Yuval–Nirkin algorithm [37]. Then, each ROI was designed to have a proportional size to the total number of pixels of the face. Of note, the number of face pixels was automatically segmented by an intensity-landmark-based algorithm purposely developed for this study. Specifically, we first separated the face from the neck and the torso using the previously estimated face contour landmarks. Then, we excluded hair and background by segmenting IR maps with a threshold-based segmentation on the pixel temperature in range (30 ${}^{\circ }$C < Tp < 38 ${}^{\circ }$C). Finally, we tracked the centres of the ROIs throughout the frames with a Matlab PointTracker object based on Kanade–Lucas–Tomasi feature-tracking algorithm [38], to properly control for potential undesired subject’s movement. For each frame, we evaluated the median temperature value of each ROI, obtaining 14 thermal signals per subject. High-frequency noise was filtered out by a moving median filter and the outliers (temperature values exceeding 3 standard deviations) were replaced by their nearest value. Then, from each thermal signal, we computed 4 features: the mean (**Mean**), the standard deviation (**Std**), and the mean and the standard deviation of the signal’s derivative (**DMean**, **Dstd**). Of note, the thermal camera automatic non-uniformity correction (NUC) was performed before the beginning of the experiment for reliability purposes. Finally, NUC was disabled during the acquisition as we observed that it corrupted the signals by introducing abrupt thermal changes [30].

#### 2.4.2. EDA Processing

The EDA signal reflects changes in the skin conductance induced by sweat glands’ activity, which is controlled by the sympathetic branch of the ANS [4]. Therefore, EDA signal processing is a powerful tool to quantify the SNS dynamics. More specifically, the EDA signal can be decomposed into a tonic and a phasic component, which are characterized by different time scales. The tonic is a slow-varying component whose spectrum is below 0.05 Hz. Conversely, the phasic reflects short-term and event-related responses. Here, we used the cvxEDA model to extract tonic and phasic components from the raw EDA signal. Such a model exploits a rigorous framework integrating Bayesian statistics, convex optimization, and sparsity, to decompose the EDA signals without the need of pre- and post-processing steps [39]. Indeed, one of the outputs of the cvxEDA algorithm is the white Gaussian noise term, which incorporates model prediction errors as well as measurement errors and artefacts. Afterwards, for each experimental session we extracted several features related to the SNS activity starting from the tonic and phasic components: i.e., the mean tonic value (**TonicMean**) and the standard deviation of the tonic (**TonicStd**) averaged within non-overlapped 20 s long time windows; the maximum peak (**PksMax**), the mean of the phasic component (**PhasicMean**), the standard deviation of the phasic component (**PhasicStd**), the number of peaks (**NPks**), the sum of peaks (**PksSum**) averaged within non-overlapped 5 s long time windows, and the **EDAsymp** (power of the SC spectrum in the range of 0.04 Hz–0.25 Hz) [40], within the entire *Rest* and *Stroop* sessions.

#### 2.4.3. HRV Processing

ECG recordings were analyzed with Kubios HRV [41]. The interbeat (RR) series were extracted from the ECG using the well-known Pan–Tompkins algorithm [42]. Algorithm-related artefacts were removed by applying the cubic spline interpolation method. Then, the obtained RR time series were resampled at 4 Hz to derive the heart rate variability (HRV) signals [43]. Starting from the HRV time series, several features were derived in the time, frequency, and nonlinear domain for both the *Rest* and *Stroop* session. Specifically, for each condition, we estimated in 3 min long segments the mean value of HRV signal (**mean HRV**), the standard deviation of HRV (**std HRV**), the square root of the mean squared differences of successive normal-to-normal (NN) intervals (i.e., **RMSSD**), the percentage number of pairs of adjacent NN intervals differing by more than 50 ms (**pNN50**), the power expressed as a percentage of total power in the low-frequency (**LF**, 0.04–0.15 Hz) and high frequency (**HF**, 0.15–0.40 Hz) ranges, the ratio of LF to HF power (**LF/HF ratio**), the minor (**SD1**) and major (**SD2**) axis of the ellipse that best fits the Poincaré plot of RR intervals, and the sample entropy (**SamplEn**).

#### 2.4.4. RESP Processing

The RESP signal was used as a further index of sympathovagal activity. Specifically, we estimated the mean frequency of respiration (**RESP freq**) for each condition and used it as a potential discriminating feature among conditions.

### 2.5. Exploratory Statistical Analysis

We performed an exploratory statistical analysis to assess at the group level how *Rest* and *Stroop* sessions differed in terms of ANS correlates. Five subjects were excluded from the analysis on EDA features and one from the thermal analysis due to the presence of artefacts related to excessive and frequent movement. Accordingly, these 6 subjects were excluded from the subsequent classification analyses. A non-parametric Wilcoxon sign-rank test was performed for each EDA, HRV, RESP, and thermal feature between *Rest* and *Stroop* sessions (α = 0.05). We controlled false discovery rate through the Benjamini–Hochberg (FDR-BH) correction for multiple hypothesis testing [44]. To evaluate the psychometric changes induced by the *Stroop* session, the same test was performed on the self-assessed stress scores recorded before and after the task.

### 2.6. Classification—Stress and Rest Recognition

We explored the contribution of thermal features in the automatic recognition of *Rest* and *Stroop* sessions. In particular, we compared three SVM models for stress classification. More specifically, we performed three supervised binary classifications using three different sets of features: a full feature-set (*Full set*) composed of features from HRV, EDA, RESP, and thermal imaging, a set without the thermal features (*No-Thermo set*) and a set exclusively made of thermal features (*Thermo set*).

Given the large number of features in our dataset compared to the number of observations, it is crucial to reduce the dimensionality of the classification problem by applying a feature selection strategy (FS). To this aim, we trained an SVM-RFE [45] model that implements an embedded FS based on recursive feature elimination (RFE) strategy. More specifically, SVM-RFE is an application of RFE using the weight magnitude as a ranking criterion and iteratively removing the feature that has the least influence on the SVM weight-vector magnitude. It is worthwhile to note that highly correlated features can bring wrong estimations of features importance in feature selection algorithms. In fact, due to the so-called correlation bias, correlated features can receive underestimated ranking criteria, with a consequently higher risk to be removed [46]. Accordingly, in our study, we adopted the SVM-RFE algorithm that implements a correlation bias correction (CBR) (see [45]). In this way, the ranking criteria is corrected for collinearity by the CBR. Embedded methods as the one implemented here allow not only maximizing the classification accuracy but also facilitating data understanding thus reducing the risk of overfitting. They are defined as embedded because the search of the optimal subset is built into the classifier construction [47]. However, if the FS is embedded into the classifier construction, then the initial number of features in the input dataset should not be comparable with or even greater than the number of observations, otherwise, this would affect the correct estimation of the SVM model weights on which the RFE is based.

For the aforementioned reason, here, we implemented a two-stage feature selection algorithm [48]: first, we reduced the initial number of features by performing a preliminary filter-type feature selection based on the correlation between each feature and the dependent variable (the class label) [49]; secondly, we applied the SVM-RFE model on the pre-filtered dataset. More specifically, in the first stage, for each feature we computed the correlation with the corresponding class (labels: 1 = *Stroop*, 2 = *Rest*) and selected the features for which correlation was significant (*p* value ≤ 0.05).

The classifiers were implemented in Matlab using the LIBSVM library. We selected a nonlinear νSVM-RFE with a radial basis function (RBF) kernel, and default parameters values. As for the out-of-sample testing, we used a leave one subject out (LOSO) cross-validation to avoid bias in the accuracy level estimation. Specifically, at each iteration, all data from all subjects were used as a training set except one subject, which was used as a test set. The choice of the subject to be excluded from the training set at each iteration was not random. Indeed, all subjects were used as testing set at some point in the iterations.

## 3. Results

**Figure 3:**
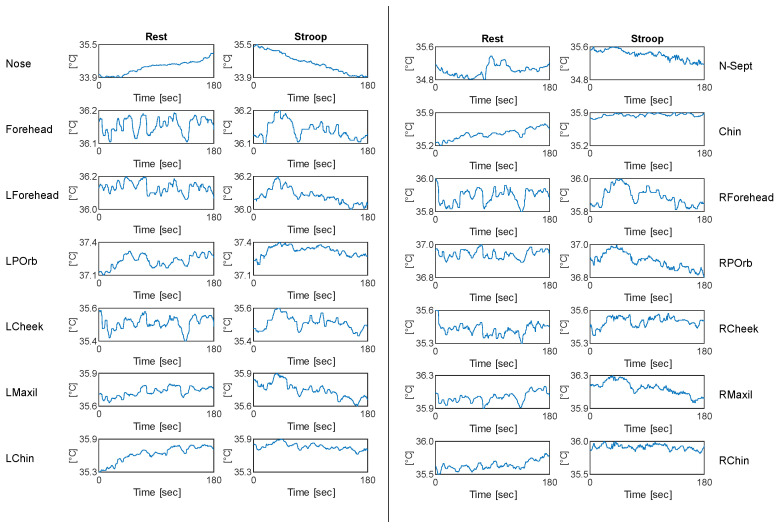
Thermal signals of one sample subject for each ROI in both rest and stress conditions. The temperature range of the plots was set equal for both conditions in each ROI, to allow visual comparison.

**Figure 4:**
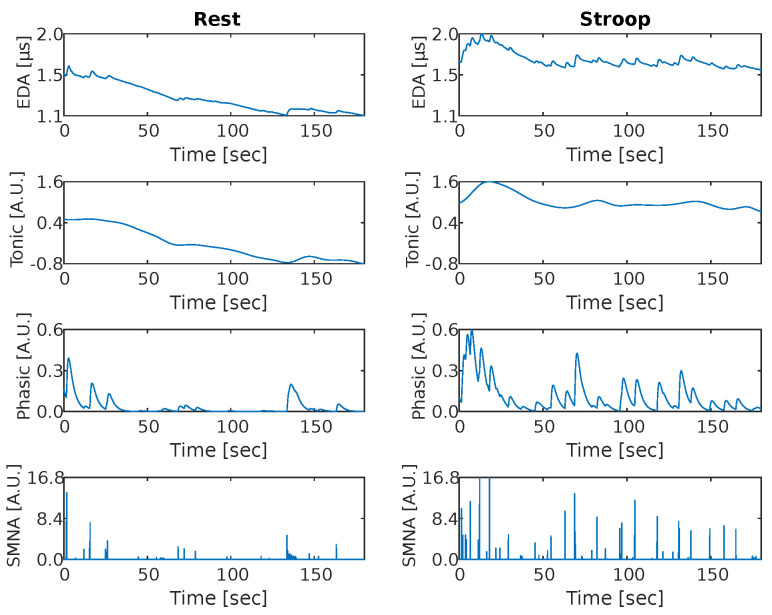
Application of the cvxEDA algorithm decomposition to the EDA signal of one sample subject in both rest and stress conditions.

An example of processed thermal signals of a sample subject for each ROI is reported in Figure 3. In Figure 4, we also report an example of the resulting signals of the cvxEDA decomposition algorithm for a sample subject.

PSS tests showed that more than 75% of the subjects were inclined to moderately feel stress. More specifically, 4 subjects out of 25 exhibited low stress, 19 moderate stress, and 2 high stress.

### 3.1. Exploratory Statistical Analyses

**Figure 5:**
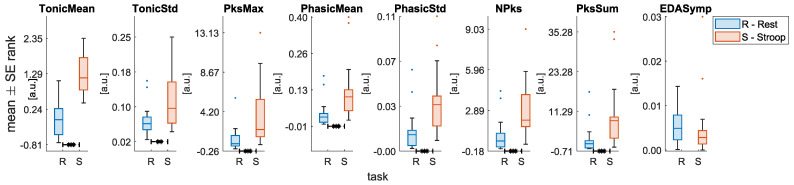
Statistical comparison for each of the EDA features extracted. Significant differences between *Rest* and *Stroop* sessions after the Wilcoxon signed rank test with FDR-BH correction are highlighted with an asterisk (* = *p* < 0.05; ** = *p* < 0.01; *** = *p* < 0.001).

The Wilcoxon test on the reported level of perceived stress indicated that subjects were more stressed after the task compared to before (median ± mad = 2 ± 1.12 vs. median ± mad = 6 ± 1.87, *p* < 0.001). Concerning the physiological data analysis, most of the features extracted from autonomic signals confirmed the expected significant difference between *Stroop* and *Rest* sessions. Specifically, except for **EDASymp**, all EDA features significantly differed between the two states, as shown in Figure 5. The significant increase of the EDA features indicates a higher activation of the SNS during the task than during rest at both tonic and phasic level.

**Figure 6:**
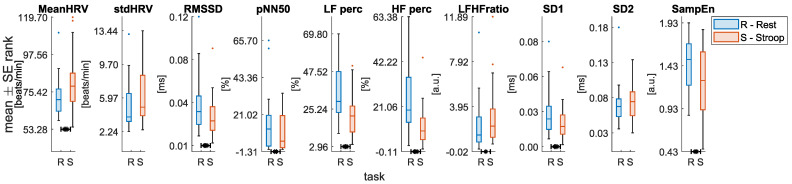
Statistical comparison for each of the HRV features extracted. Significant differences between *Rest* and *Stroop* sessions after the Wilcoxon signed rank test with FDR-BH correction are highlighted with an asterisk (* = *p* < 0.05; ** = *p* < 0.01; *** = *p* < 0.001).

**Figure 7:**
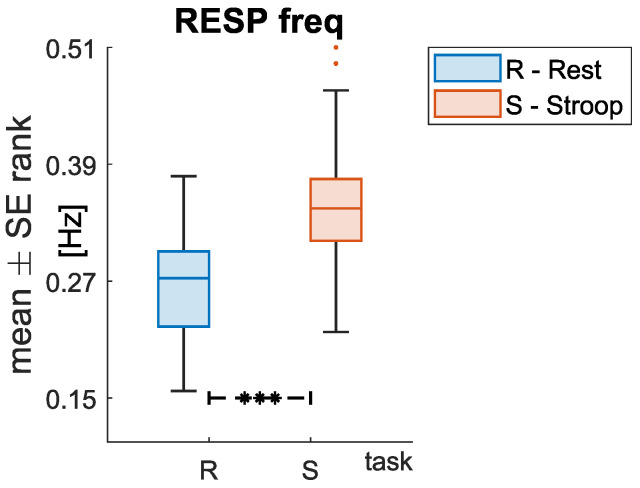
Statistical comparison for the respiratory feature extracted. Significant differences between *Rest* and *Stroop* sessions after the Wilcoxon signed rank test with FDR-BH correction are highlighted with an asterisk (* = *p* < 0.05; ** = *p* < 0.01; *** = *p* < 0.001).

Moreover, all the HRV related features showed a significant variation during *Stroop* compared to *Rest* sessions, with the only exception of **stdHRV** and **SD2** (Figure 6). Specifically, **MeanHRV** and the **LF/HF ratio** increased during the *Stroop* compared to the *Rest* session. On the other hand, the remaining features decreased during the *Stroop* session. Among these, a variation of interest is the reduction in HF power during the *Stroop* session, considering its relation to PNS activity. Conversely, the respiratory frequency increased significantly during the *Stroop* compared to the *Rest* session, as shown in Figure 7.

**Figure 8:**
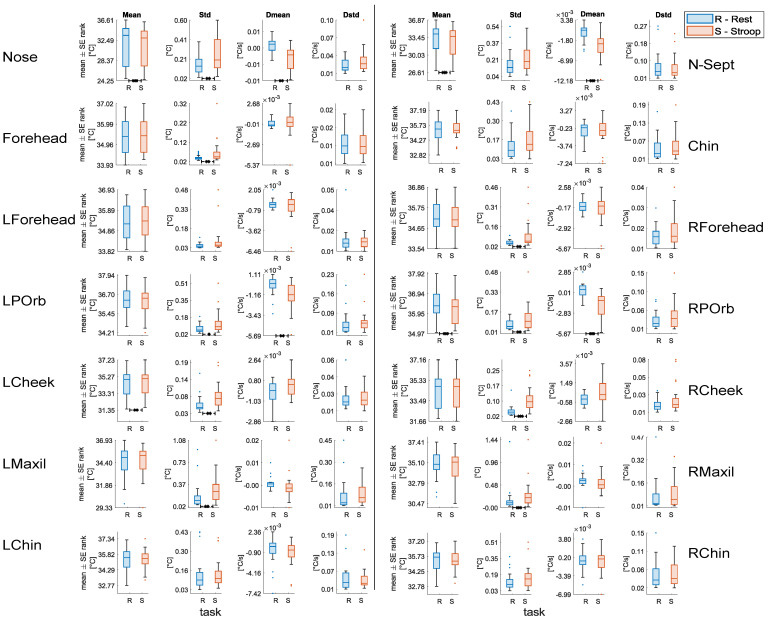
Statistical comparison for each of the thermal features extracted. Significant differences between *Rest* and *Stroop* sessions after the Wilcoxon signed rank test with FDR-BH correction are highlighted with an asterisk (* = *p* < 0.05; ** = *p* < 0.01; *** = *p* < 0.001).

The results of the statistical analysis performed on the thermal features are shown in Figure 8. Among the considered ROIs, L-Forehead, Chin, L-Chin, and R-Chin did not show any significant alteration between sessions. Conversely, the remaining ROIs, except for N-Sept, showed a significant increase in the **Std** during the *Stroop* session. On the other hand **Mean** and **DMean** of Nose, N-Septum, and R-POrb decreased significantly during the *Stroop* session. Similarly, **DMean** of L-POrb showed a significant decrease during the *Stroop* session. On the contrary, **Mean** of L-Cheek increased significantly during the *Stroop* compared to the *Rest* session. Finally, **DStd** did not show any significant variation between the tasks.

### 3.2. Classification

**Figure 9:**
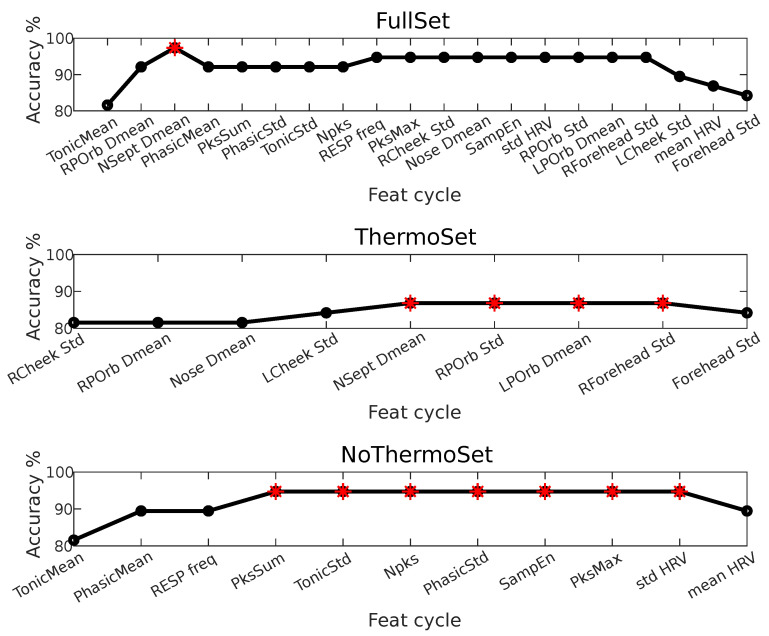
Accuracy trend of stress/non-stress recognition problem as a function of the selected features. The features are ranked according to the SVM-RFE criterion. Maximum accuracy is highlighted by red asterisks.

After the preliminary filter-type feature selection, *Full Set* dimensionality was reduced to 20, *Thermo Set* to 9, and *No-Thermo Set* to 11, as reported in Table 1. On these three different sets, we compared the outcomes of the SVM-RFE classifier. Specifically, for each set, we evaluated the classification accuracy by means of LOSO cross-validation. The average accuracy at each cycle is reported in Figure 9. In particular, we observed a maximum accuracy of 97.37% for the *Full set* and 94.74% for the *No-Thermo set*, whereas for the *Thermo set* we observed a maximum accuracy of 86.84%. In particular, the peak of the accuracy trend for the *Full Set* was obtained with two thermal features and one EDA feature. The accuracy peak in the *Thermo Set* was obtained when the first five most relevant features, according to the RFE criterion, were considered. In the *NoThermo Set*, the maximum the accuracy was reached when four features were considered (three EDA features and the respiratory frequency).

In Table 1, for each set, we report the results of the filter-type feature selection procedure, along with the feature ranking according to RFE. We observed that within the *Full set*, nine out of twenty features were thermal ones and two of them (i.e., RPOrb **DMean**, N-Sept **DMean**) ranked among the first three features. In the *No-Thermo set*, all EDA features except **EDAsymp** were selected, together with **RESP freq**, **mean HRV**, and **std HRV**. Notably, the **TonicMean** was ranked as first in both *Full set* an *No-Thermo set*. In the *Thermo set*, RCheek **Std** was the first ranked feature, with an accuracy higher then 80%. The same accuracy was reached in the other two feature sets when considering only **TonicMean** from the EDA signal. Finally, in the *Thermo set*, several ROIs were selected (Table 1). In particular, only features based on signal derivative mean (i.e., **DMean**) and signal standard deviation (i.e., **Std**) were included.

For each feature set, we report the confusion matrices related to the subset that achieved the maximum accuracy (Table 2). For the *Thermo set*, we observed a percentage of false negatives (error type II) of 15.79%, lower values were found in the *Full set* and in the *No-Thermo set*.

## 4. Discussion

In this study, we explored the possibility of using the thermal camera for automatic acute stress detection either in combination with other typical physiological signals or alone. In this latter condition, we made a relevant step towards a reliable contactless subject-independent stress recognition system exclusively based on thermal features. This might contribute to overcoming issues given by multiple wearable devices and support the shift towards contactless stress monitoring.

We designed a computerized and paced version of the Stroop test to mimic a more realistic stressful situation [50,51]. This allowed to reduce the interaction of the subjects with the experimenter, to standardize the external stimuli for all the participants, and to prevent speech artefacts in the signals. On the other hand, the use of well-established measures for stress recognition allowed us to confirm the effectiveness of the stimulation protocol. Here, we observed a significant increase in self-assessed perceived stress levels after the *Stroop* session, as expected. Furthermore, the group-level preliminary statistical analyses on physiological signals supported this observation, as significant variations of most of the physiological parameters taken into consideration were observed. Among the HRV related features, we observed a decrease in **RMSSD**, **pNN50**, and **HF** which are likely to reflect a decrease in the PNS activity [52,53]. Moreover, we observed an increase of the **LF/HF ratio** which was previously associated with psychological stress due to changes in the sympathovagal balance [54]. To study the effect on the SNS activity, we based on the analysis of the EDA. Indeed, many of these features were found to be particularly suitable for stress monitoring [10,55]. In our study, we observed an increase in the mean value of the tonic component (**TonicMean**) during the *Stroop* session, which is likely to reflect the overall increase of the subject’s arousal level. Furthermore, the increase of phasic component features indicated a more frequent and stimulus-related SNS activation, which may be related to the repetitive task-induced request for attention. Finally, we observed a higher respiratory frequency during the *Stroop* session, possibly reflecting sustained attention and mental stress [56].

The preliminary statistical analysis performed on thermal features allowed us to distinguish among rest and stress conditions as well, with the main advantage of providing the measures of interest in a contactless fashion. Stressful conditions were found to trigger a sympathetically driven vasoconstriction of the blood vessels in the skin [19,20,27,57], which is reflected by a drop in the temperature of the tip of the nose [22,24,58]. In our study, we observed such temperature drop as well. Additionally, our results extend such significant variations also to other facial regions, i.e., forehead, cheeks, chin, periorbital, and maxillary areas. In particular, we found an increase in the mean temperature of the forehead during Stroop, in agreement with [29], and which may be associated with the activation of the corrugator muscle in response to stress [19]. Moreover, we observed a significant temperature increase in the left cheek, which could be due to the well-known blushing phenomenon due to embarrassment [59] and possibly associated with social stress. It is worthwhile noting that some of these ROIs did not show a coherent thermal pattern throughout the literature [60]. In this view, although further investigation will be needed, we can confirm that several of these ROIs were informative for stress/non-stress recognition by means of the SVM-RFE algorithm used in this work.

We evaluated the contribution of thermal features in a stress recognition task by comparing the performance of a classifier trained with and without thermal features (i.e., *Full set* vs. *No-Thermo set*). Furthermore, we evaluated the ability of the thermal features alone to recognize stress (i.e., *Thermo set*).

We built a classification of stress at the single-subject level [61], avoiding possible bias introduced by other validation strategies such as the K-fold ones (when data from the same subject is presented both in the training and validation sets). Our results showed a significant contribution given by the features derived by the thermal signal which increased the accuracy from 94.74% to 97.37% (*Full Set*). Moreover, the peak of accuracy was obtained with only three features, two of which extracted by the thermal signal (i.e., **DMean** of RPOrb and N-Sept).

Notably, the classification exclusively based on thermal features achieved a maximum accuracy of 86.84%. Although this value was lower than the one obtained with wearable devices, our result suggests that stress recognition by means of contactless thermal imaging can still achieve good results. In this view, it is worthwhile noting that stress recognition based only on thermal imaging would have the advantages of being more comfortable with respect to intrusive contact-based systems.

To minimize the risk of overfitting due to the limited number of observations, we decreased the complexity of the model, maximizing the classification accuracy. In particular, we implemented a two-stage feature selection (FS) strategy to reduce the dimensionality of the classification problem. First, we reduced the number of features by performing a preliminary filter-type feature selection based on the correlation between each feature and the dependent variable (i.e., the class label) [49]. Moreover, we applied the SVM-RFE +CBR algorithm on the reduced dataset [45]. This is an embedded FS that not only reduces the risk of overfitting, but also implements a correlation bias reduction strategy. Moreover, it has been proved that embedded FS methods outperform the filter and wrapper approach, scoring features’ importance based on the output of the predictive model [62,63,64]. Finally, we evaluated generalization capabilities through leave-one-subject-out (LOSO) cross-validation strategy which made our results unbiased and subject independent.

The feature selection step allowed to highlight the most important features for optimal classification. Many thermal features were selected for the classification when using the *Full Set*. Notably, 9 features out of 20 were thermal ones. It is worthwhile noting that among the thermal features only the standard deviation **Std** and the mean of the derivative **DMean** were selected (both of them being measures of thermal variation). Specifically, **Std** is a measure of the amount of variation in temperature, while **DMean** is an index of the velocity of temperature variations. This result, in line with other studies [65], suggests that absolute temperature is of less interest compared to its relative changes. Other previous studies have tested the performance of combined or singleton wearable and contactless systems [66,67,68], with lower accuracy [67] or showing some limitations in the methodological approach [66,68]. Whereas the authors in [68] based the classification on respiratory features extracted from the thermograms, regardless of strictly-thermal signatures, another study [66] employed a decision tree classifier with LOSO validation to investigate whether feature fusion of the physiological and thermal modalities could further improve the acute stress detection rate. Despite their good accuracy, our processing and classification pipeline was able to outperform the decision tree classifier.

A potential limitation of psychophysiological studies involving thermal imaging is related to the confounding factors due to the surrounding environment. Infrared technology is highly sensitive to reflected radiation from nearby objects. In this work, acquisitions were performed in a controlled environment (e.g., no direct ventilation on the subjects and reasonable distance from the window). Furthermore, we prevented the subject’s thermoregulation processes by controlling for room temperature and humidity to be steady and at a comfortable level [69]. Indeed, these processes could hide the small thermal variation due to the psychological factors under consideration. In addition, we have ensured an acclimatization period prior to the experiment of 10 min, as suggested in [70], to allow for skin temperature patterns stabilization [36,71]. Accordingly, we can assume that the observed physiological changes are due to the SNS/PNS balance in response to emotional stress rather than to other phenomena. Finally, another limitation is given by the movement constraints that are needed to ensure low ROI tracking errors and therefore low noise in the thermal signals. Some studies use a chin rest to avoid sudden head rotation or direction change [72]. Here, we asked the subject to remain still during the acquisitions. Afterwards, we visually inspected the signals and removed subjects with irreducible artefacts. However, further studies will try to investigate a method able to mitigate these limitations.

It is worthwhile to mention that different kinds of stimuli may induce stress (e.g., emotional, physical). In this work, we used only one type of mental stressor, obtained by means of the well-known Stroop test. However, future studies will consider more complex protocols (e.g., by including different stressors) to extend the results to different kinds of stress. Other insights could include thermal feature extraction also in the frequency or non-linear domains, possibly improving the performances of the classifier. Finally, in the perspective of extending these results to clinical uses, the developed system could be tested also with pathological subjects.

## Figures and Tables

**Table 1:** Feature ranking according to the RFE criterion.

Rank	*Full Set*	Rank	*Thermo Set*	Rank	*No-Thermo Set*
1	TonicMean	1	RCheek Std	1	TonicMean
2	RPOrb Dmean	2	RPOrb Dmean	2	PhasicMean
3	N-Sept Dmean	3	Nose DMean	3	RESP freq
4	PhasicMean	4	LCheek Std	4	PksSum
5	PksSum	5	N-Sept DMean	5	TonicStd
6	PhasicStd	6	RPOrb Std	6	NPks
7	TonicStd	7	LPOrb DMean	7	PhasicStd
8	Npks	8	RForehead Std	8	SampEn
9	RESP freq	9	Forehead Std	9	PksMax
10	PksMax			10	std HRV
11	RCheek Std			11	mean HRV
12	Nose DMean				
13	SampEn				
14	std HRV				
15	RPOrb Std				
16	LPOrb DMean				
17	RForehead Std				
18	LCheek Std				
19	mean HRV				
20	Forehead Std				

**Table 2:** Confusion matrices. N-S: non-stress; S: stress. The percentage of true positives and true negatives of each class is represented in the diagonal of each table.

		Predicted Classes					
		**S**	**N-S**	**S**	**N-S**	**S**	**N-S**
	S	94.74%	5.26%	84.21%	15.79%	100%	0%
Actual Classes	N-S	0%	100%	10.53%	89.47%	10.53%	89.47%
		*Full Set*		*Thermo Set*		*No-Thermo Set*	

## Data Availability

The data are not publicly available due to privacy restrictions.
